# Socially Responsible Behaviors of Nursing Students in Private Universities in Santiago, Chile: A Study on the Alignment with Sustainable Development Goals

**DOI:** 10.3390/nursrep15030093

**Published:** 2025-03-10

**Authors:** Sandra Vera-Ruiz, Alejandro Vega-Muñoz, Nicolás Contreras-Barraza, Diego Silva-Jiménez, José A. Iturra-González, Ana Martín-Romera

**Affiliations:** 1 Vicerrectoría Académica, Universidad Autónoma de Chile, Santiago 8910123, Chile; sandra.vera@uautonoma.cl; 2 Centro de Investigación en Educación de Calidad para la Equidad, Universidad Central de Chile, Santiago 8330507, Chile; 3 Facultad de Ciencias Empresariales, Universidad Arturo Prat, Iquique 1110939, Chile; 4 Pontificia Universidad Católica de Valparaíso, Valparaíso 2340025, Chile; 5 Facultad de Medicina y Ciencias de la Salud, Universidad Central de Chile, Santiago 8330507, Chile; diego.silva@ucentral.cl; 6 Escuela de Medicina, Facultad de Ciencias Médicas, Universidad de Santiago de Chile, Santiago 9170022, Chile; alejandro.iturra.g@usach.cl; 7 Facultad de Ciencias de la Educación, Universidad de Granada, 18011 Granada, Spain; amromera@ugr.es

**Keywords:** social responsibility, nursing education, sustainability competence, higher education, competence development, skills, nursing

## Abstract

**Background/Objectives**: Universities, particularly in nursing education, have evolved to incorporate University Social Responsibility (USR) into their curricula, emphasizing ethical, social, and sustainable competencies, which align with the Sustainable Development Goals (SDGs) to enhance healthcare and education. The study examines socially responsible behaviors, ethical competencies, and sustainability values among nursing students in private universities in Santiago, Chile, and their relation to sociodemographic variables. **Methods**: The study measures social responsibility in nursing students at private universities, accredited and co-financed by the State in Santiago, Chile, using the ICOSORE-U questionnaire, with statistical analyses such as EFA and CFA to validate the scale and evaluate correlations with sociodemographic variables. **Results**: The study validated the ICOSORE-U-10 scale for social responsibility in Chilean nursing students through both exploratory and confirmatory factor analysis. The results revealed a two-factor structure and an acceptable internal reliability (α = 0.841), with no significant differences related to sociodemographic variables. The findings confirmed that socially responsible behaviors are positively correlated with the development of ethical competencies and sustainability among these students. **Conclusions**: the socially responsible behaviors of nursing students are mainly determined by their orientation towards University Social Responsibility (USR) and not by sociodemographic variables. This orientation favors the development of ethical and professional competencies, improving their ability to address social and ethical challenges in the health field. Integrating the principles of USR in university education is key to training professionals committed to social justice, equity and sustainability in health care.

## 1. Introduction

Universities have the mission of training competent professionals who assume leadership roles and contribute significantly to social welfare and community development [1,2]. In this context, their role has evolved beyond traditional teaching and research, incorporating university social responsibility (USR) as a key dimension that links academia with contemporary social needs [3,4]. This transformation is particularly relevant in nursing education, where training must prepare students to address challenges related to health, social equity, and sustainability.

USR is defined as the university’s ability to generate and apply ethical values and social principles through management, teaching, research, and outreach [2]. This approach aligns with the Sustainable Development Goals (SDGs), particularly SDG 3 (Good Health and Well-being) and SDG 4 (Quality Education), thereby promoting equity in healthcare and ensuring comprehensive nursing education [5,6]. In Chile, the Metropolitan Region accounts for approximately 60% of nursing students [7], which underscores the importance of investigating how USR influences their training and the need to integrate this component into academic programs [8].

In this regard, the implementation of innovative methodologies—such as the integration of the 4Ps (pathophysiology, pharmacology, physical/health assessment, and health promotion) in master’s-level nursing education [9] and the use of design thinking pedagogies to enhance innovative competencies [10]—serves as an example of how to foster the holistic development of students. Moreover, approaches such as problem-based learning [11] and team-based learning [12] contribute to developing transversal skills, including leadership, critical thinking, and social responsibility [1,13].

The ethical commitment in nursing extends beyond the acquisition of technical skills. It is essential that professionals demonstrate ethical and moral awareness in their daily practice, a requirement that is critical for addressing the complexities of healthcare and preventing moral distress in high-pressure environments [14]. Training in ethics and the promotion of dignity within the academic setting—such as the discussion surrounding alternatives to the implementation of “trigger warnings” [15]—are crucial components in preparing professionals committed to health and social well-being.

The incorporation of innovative technologies and pedagogical strategies also plays a decisive role in nursing education. For instance, the use of digital tools, such as mobile applications for learning basic physical assessment [16] or interactive virtual environments for teaching pathophysiology [17], demonstrates how the integration of technology can enhance learning and digital competence in health [18]. Similarly, the incorporation of electronic medical records [19] and the use of open educational resources [20] are strategies that facilitate access to information and promote the continuous updating of curricular content.

Innovative curricular design that incorporates methodologies such as the flipped classroom [21] and gamification approaches [22] has been shown to improve educational experience and the acquisition of competencies. Initiatives that combine traditional teaching with new learning tools—such as the creation of online learning environments [23] and the use of theoretical frameworks for curriculum design [24]—allow education to be transformed into communities of practice that reflect the diversity and challenges of today’s context.

Competency development has also extended to complementary areas, such as the development of sexual competence in multicentric settings [25] and the strengthening of intercultural competence through academic exchange programs [26]. Furthermore, studies examining the experiences of nursing educators with students from diverse cultural backgrounds underscore the necessity of adapting training to address the diversity present in the classroom [27].

Additionally, the assessment and measurement of competencies through validated instruments enable the identification of strengths and areas for improvement within teaching-learning processes, thereby consolidating an evidence-based approach for decision-making in educational management [28]. Similarly, studies that analyze the outcomes of concept-based curricula [29] and the effects of pedagogical strategies on knowledge translation [23] reinforce the importance of implementing educational models that meet the current demands of the healthcare environment.

Based on the literature review, it is recognized that nursing education should extend beyond the development of technical competencies, incorporating the ethical training of students. This aspect is crucial for future professionals to act as agents of change in their communities, promoting social well-being and sustainability in the healthcare field. In this context, the need arises to explore how the socially responsible behaviors of nursing students relate to their development in ethical competencies and sustainability. Integrating these behaviors into academic training could be critical to ensure that healthcare professionals are better prepared to face current social and environmental challenges. Therefore, the research questions guiding this study are: What are the factors that define the socially responsible behaviors of nursing students in private universities in Santiago (Chile)? How do sociodemographic variables influence the socially responsible behaviors of nursing students, and how do these behaviors influence their professional preparation?

A competency-based educational model is fundamental to achieving the objectives of integrating University Social Responsibility (USR) into nursing education in Chile, aligned with the SDGs of health and quality education. Nursing education should focus on developing technical, social, and ethical competencies that enable future professionals to perform effectively in diverse contexts [1,30,31].

The study aims to analyze socially responsible behaviors, linked to the development of ethical competencies and sustainability values, of nursing students in private universities in Santiago (Chile), as well as the relationship of these behaviors with sociodemographic variables. The ICOSORE-U scale is used to identify the key factors that influence the formation of professionals committed to responsible and sustainable healthcare practices, and to know the related sociodemographic variables.

## 2. Materials and Methods

### 2.1. Participants

The measurement of social responsibility in nursing students at private universities (universities which are members of the Corporation of Private Universities), with quality accreditation and ascribed to the state system tuition-free universities in the Metropolitan Region of Santiago (Chile) was conducted. In Chile, undergraduate nursing has a high degree of femineity and among private universities with quality accreditation and ascribed to the state system tuition-free, it reaches 81% of female enrollment, and the internationalization of nursing students in this type of universities is low, with 98% of the students being Chilean nationals [32].

### 2.2. Context

Chile is a country in constant evolution, where modernity coexists with its deep cultural roots. With a population of 18.6 million inhabitants, most live in cities, especially in Santiago, reflecting its growing urbanization. Its society is diverse, with an identity marked by indigenous (Mapuche 9.1%, Aymara 0.7%, other Indigenous groups 1%) and European heritage, and a common language, Spanish, that unites its inhabitants. Religion also plays a role in shaping its cultural landscape, with 42% of the population identifying as Roman Catholic, 14% as Evangelical, 6% following other religions, and 37% having no religious affiliation. Despite advances in education, health, and the economy, inequality remains a challenge, impacting access to opportunities. With an aging population and a declining birth rate, the country faces the challenge of ensuring the well-being of its citizens while remaining an attractive destination for immigrants seeking a better future [33].

### 2.3. Instruments

The Chilean questionnaire Inventory of Socially Responsible Behaviors in University Students (*Inventario de Conductas Socialmente Responsables en Universitarios*, ICOSORE-U) is used as the base measurement instrument [34]. The survey was applied with a 5-point Likert scale: Strongly Disagree = 1, Disagree = 2, Neither Disagree nor Agree = 3, Agree = 4, and Strongly Agree = 5. The questionnaire has been self-administered online, with a prior informed consent response, collecting the survey without a possible identification of the respondent and presenting only non-potentially identifiable human data.

### 2.4. Data Analysis and Processing Procedures

The research aim established in the previous section has been accompanied by two types of hypotheses, which are detailed below.

Hypothesis of the factors:

**H_01_:**  *There is no significant relationship between socially responsible behaviors of nursing students and their development of ethical competencies and sustainability in private universities in Santiago, Chile.*

**H_1_:**  *There is a significant relationship between the socially responsible behaviors of nursing students and their development of ethical competencies and sustainability in private universities in Santiago, Chile.*

Hypothesis of the correlations:

**H_02_:**  *(Null hypothesis): There is no significant correlation between the sociodemographic variable and the ICOSORE-U values, in nursing students at private universities in Santiago, Chile.*

**H_2_:**  *(Alternative hypothesis): There is a significant correlation between the sociodemographic variable and ICOSORE-U values, such that ICOSORE-U scores would vary as individuals’ sociodemographic variables vary, in nursing students at private universities in Santiago, Chile.*

Thus, the 7 alternative hypotheses are formalized as follows:

**H_2A_:**  *(Alternative hypothesis): There is a significant correlation between age and ICOSORE-U values, such that ICOSORE-U scores vary with the age of nursing students in private universities in Santiago, Chile.*

**H_2B_:**  *(Alternative hypothesis): There are significant differences in ICOSORE-U values between males and females, indicative of a possible correlation with gender of nursing students in private universities in Santiago, Chile.*

**H_2C_:**  *(Alternative hypothesis): The presence of children significantly influences ICOSORE-U values, differing from those without children between the nursing students in private universities in Santiago, Chile.*

**H_2D_:**  *(Alternative hypothesis): There is a significant relationship between the number of children and ICOSORE-U values, such that a higher number of children is associated with a change in ICOSORE-U values between the nursing students in private universities in Santiago, Chile.*

**H_2E_:**  *(Alternative hypothesis): Educational gratuity has a significant positive effect on ICOSORE-U values, reflecting an improvement in social and educational well-being.*

**H_2F_:**  *(Alternative hypothesis): There is a significant relationship between nationality and ICOSORE-U values, with possible differences in values depending on the nationality of the nursing students in private universities in Santiago, Chile.*

**H_2G_:**  *(Alternative hypothesis): Belonging to native peoples is associated with a significant decrease in ICOSORE-U values, reflecting a possible disparity in access to resources and services between the nursing students in private universities in Santiago, Chile.*

Using SPSS version 23 software (IBM, New York, NY, USA), the 25 items of the ICOSORE-U were analyzed by evaluating their psychometric properties [35]. First, a univariate descriptive statistical analysis was executed, focusing on variance (>0), skewness (|≤1|), and kurtosis (|≤1|) [36]. Although considering the univariate results presented by Boero et al. [34], we find it necessary to give a slack of decimal points over 1, especially to the kurtosis in which we will allow a maximum of 1.8 [37].

To measure confidence levels, the authors applied the measurement of sampling adequacy (MSA) by anti-image correlation matrix (eliminating the variable with the lowest value on the diagonal of the matrix) [38], and Kaiser–Meyer–Olkin (KMO) measure of sampling adequacy. Furthermore, the authors used Bartlett’s test of sphericity to identify items that belonged to factors within the scale as a form of exploratory factor analysis (EFA) with extraction method unweighted least squares (ULS), and rotation method Oblimin with Kaiser normalization [39], identifying through EFA the underlying structure of the data at the factor level, as a preliminary step to conducting a more structured analysis.

The authors then analyzed the exploratory factors using confirmatory factor analysis (CFA) developed with FACTOR software [40], with polychoric correlation using Hull’s method and Robust Unweighted Least Squares (RULS) [41] and Rotation Normalized Direct Oblimin, revising the Measure of Sampling Adequacy (MSA) [42], and choosing a set of factors that feature high communalities, strong factor loadings relative to the sample size, and a minimal number of items per factor (MIF) [43,44,45]. Reporting the indicators detailed in Table 1 [46]: Chi-square ratio/degree of freedom (χ^2^/df), root mean square error of approximation (RMSEA), adjusted goodness-of-fit index (AGFI), goodness-of-fit index (GFI), comparative fit index (CFI), non-normed fit index (NNFI), and root mean square root of residuals (RMSR) [45]. Thus, the CFA, based on the results of the EFA, makes it possible to confirm whether a theoretical factor structure fits the data well, verifying whether the proposed theoretical model of factors has empirical validity.

In addition, the internal reliability of the resulting instrument will be validated by calculating Cronbach’s Alpha using SPSS 23 software [47,48].

**Table 1:** Validation and reliability parameters.

Sample	Level	MIF	χ^2^/df	RMSEA	AGFI	GFI	CFI	NNFI	RMSR
≥200	Good fit	NR	≥0	≤0.05	≥0.90	≥0.95	≥0.97	≥0.97	<0.05 ++
			≤2		≤1.00	≤1.00	≤1.00	≤1.00	
	Acceptable fit	≥3	>2	>0.05	≥0.85	≥0.90	≥0.95	≥0.95	≥0.05
			≤3	≤0.08	<0.90	<0.95	<0.97	<0.97	≤0.08 ++
1935	Boero et al. [34]	4	4.11	0.057	NR	NR	0.96	NR	NR

NR: not reported. ++ indicated in Kalkan et al., 2016 [48].

Finally, once the empirical validity of the factors has been proven by the CFA, the resulting score of the ICOSORE-U scale will be obtained, to be analyzed in contrast with some relevant sample characteristics, identifying possible differences that affect socially responsible behavior results. Thus, the weighted social responsibility levels, given the latent factors, resulting from the evaluation of the students, will be analyzed by means of cross-tabulations between social responsibility and the variables: Age, Gender (GEN), Children (CHI) and Number of children (NCH), Educational gratuity (GRAT), Nationality (NAT), Native people (NPE), using Kendall’s Tau-C and Goodman–Kruskal’s Gamma tests to measure their symmetrical association [49,50].

### 2.5. Ethical Procedures

This stage of data collection was performed under informed consent (see Informed Consent Statement) and the research was carried out in accordance with that declared in the Institutional Review Board Statement.

## 3. Results

### 3.1. Sample Characterization

The social responsibility scale for university students (ICOSORE-U) was applied in the first academic semester 2023 to a set of 337 effective participants (≥200, overcoming small sample sizes for factorial analysis) [51], nursing university students from the Metropolitan Region of Chile, which concentrates about 40 percent of the national and nursing university population [52,53] and characterized as shown in Table 2.

### 3.2. Validation by Exploratory and Confirmatory Factor Analysis

First, the possible prevalence of the factors identified by Boero et al. [34] in university teachers in Chile was investigated. These five original factors address social responsibility in: Social Assistance (SA), Care Respect (CR), Cultural Citizenship (CC), Religion (RE), and Study Work (SW). When performing the univariate descriptive statistical analysis, none of the ordinal variables showed a variance of zero, indicating that they all contribute to the common variance. However, some variables present drawbacks in terms of skewness and kurtosis, as detailed in Table 3.

Thus, only 15 reported items can be considered in the exploratory factor analysis (EFA). These are subjected to anti-image correlation analysis, considering the data on the main diagonal of the matrix. (See Table 4, data highlighted in gray).

Therefore, CR7 is eliminated, and the exploratory factor analysis is readjusted with 14 variables. This new adjustment incurs the loss of CC5 (loading less than 0.4); therefore, a final exploratory readjustment with 13 variables is performed.

Table 5 and Table 6 show the unrestricted result of the exploratory factor analysis preserving the 13 variables and determining with SPSS 23 a KMO of 0.831 and Bartlett’s test with a Chi-square of 1318.641 with 78 degrees of freedom and a significance level of 0.000 for the three factors of the ICOSORE-U instrument. We obtained 54.591% of the variance explained. Although these results are viewed positively, it is noted that factor 3 does not meet the suggested minimum number of variables per factor (≥3) [42,43,51], which prompts us to explore a new option for variable reduction.

The authors also performed a confirmatory factor analysis (CFA) on the data set composed of 13 variables, using FACTOR software. The Measure of Sampling Adequacy (MSA) [41] suggests eliminating items CC1, CC3, and CC4 since their confidence intervals (at 90%) present minimum values of less than 0.5. At the same time, the loss of these three items reduces the number of factors from three to two.

Then, the CFA applied for a sample of 337 (216 excluding missing data) obtained a good KMO (Kaiser–Meyer–Olkin) equal to 0.84858 (>0.8) and Bartlett’s test of sphericity equal to 1670.2 with 45 degrees of freedom and a significance level of 0.000010. Those results are significant and good enough to present the adequacy of the polychoric correlation matrix (see Table 7).

The authors then reduced the ICOSORE-U questionnaire in terms of its latent variables into two factors (see Table 7), using the Hull method, implemented by performing an adequacy of the polychoric correlation matrix.

Table 8 sets out the proposed model results in comparison with the resulting validity and reliability values in Boero et al., 2020 [34], for the RMSEA, AGFI, GFI, CFI, and RMSR indicators by FACTOR software. In comparative terms, the proposed model reports χ^2^/df, RMSEA, CFI, and NNFI (TLI) with a good fit, better than Boero et al., 2020 [34]. Additionally, the proposed model reports AGFI, GFI, NNFI, and RMSR with a good fit, whereas Boero et al. [34] did not report these parameters.

**Figure 1:**
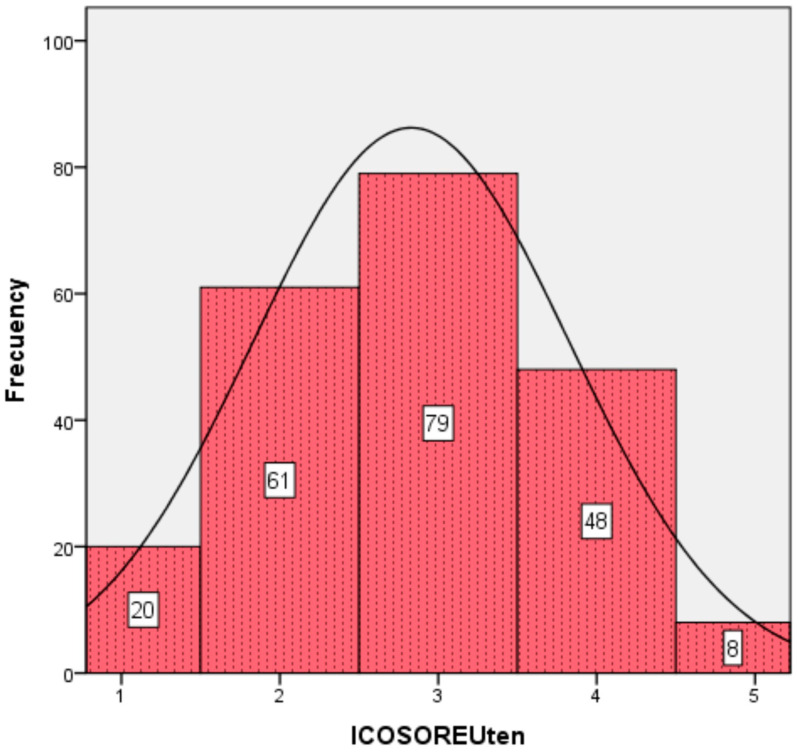
Histogram of ICOSORE-U-10 scale.

Finally, Table 9 shows the instrument’s internal reliability by SPSS 23 software, with a total Cronbach’s Alpha of 84.1% for the set of 10 items, and Figure 1 presents a histogram of the resulting scale with 216 valid cases out of 337, a mean 2.83, and good univariate parameters of variance (0.998 > 0), skewness (|0.011| ≤ 1), and kurtosis (|−0.544| ≤ 1).

Consequently, it is possible to assert that the proposed theoretical model of the factors has empirical validity.

### 3.3. Resulting Scale Analysis

The resulting scale with 10 items (ICOSORE-U-10) is analyzed using means of cross-tabulations between social responsibility and the variables: Age, Gender (GEN), Children (CHI) and Number of children (NCH), Educational gratuity (GRAT), Nationality (NAT), Native people (NPE), using Kendall’s Tau-C and Goodman–Kruskal’s Gamma tests to measure their symmetrical association [49,50]. As an outcome of this analysis, the resulting scale does not present statistically significant differences for the diverse values of the characterization variables in the sample studied (See Table 10).

To conclude Section 3, the statistical analyses conducted in this study allowed testing the proposed hypotheses. The null hypothesis (H_0_), which stated that there was no significant relationship between nursing students’ socially responsible behaviors and their development of ethical competencies and sustainability, was rejected. The alternative hypothesis (H_1_), which suggested that there was a significant relationship between these factors, was accepted. With a *p*-value of 0.001, the results confirmed that socially responsible behaviors are positively correlated with the development of ethical competencies and sustainability in nursing students. This finding emphasizes the importance of integrating social responsibility into nursing education programs to promote comprehensive training that prepares future professionals to face the ethical and social challenges in their practice. Additionally, it is possible to assert that the ICOSORE-U-10 scale does not present statistically significant differences for the diverse values of the sociodemographic characterization variables in the sample studied: Age, Gender, Children, Number of children, Educational gratuity, Nationality, and Native people.

## 4. Discussion

The results of this study highlight the importance of University Social Responsibility (USR) in the education of nursing students, particularly in relation to the development of ethical values and competencies linked to sustainability. In a context in which higher education institutions play a key role in the preparation of professionals committed to health equity, the measurement and analysis of these dimensions are essential to understand their impact on academic and professional training. Several authors [4,5,6] argue that USR not only contributes to the individual training of students, but also transforms the institutional culture of universities, creating more inclusive and sustainable learning environments. The results of this study reinforce this position, demonstrating that students with higher levels of USR not only internalize values of equity and social responsibility, but also show a greater commitment to sustainability in healthcare.

Higher education plays a crucial role in developing generic competencies such as critical thinking and responsible citizenship [1,31]. These competencies are essential for fostering ethical reasoning and social commitment among students. In this sense, the findings of our research align with the emphasis on higher education as a space for fostering ethical analysis and decision-making based on principles of social justice. The results of our study confirm the relevance of USR as a transversal axis in nursing education, showing that its development is linked to a greater capacity for ethical analysis and decision-making based on principles of social justice.

Through the application of the ICOSORE-U scale, this study identified that USR is manifested in various dimensions, such as social assistance, respect for diversity, academic responsibility, and cultural citizenship. These factors are not only part of students’ university experience but also influence their ability to address ethical dilemmas and make informed decisions in their professional practice. Active learning methodologies, such as Problem-Based Learning (PBL), play a crucial role in nursing education, contributing to the development of autonomy, clinical reasoning, and communication skills [11]. Integrating these approaches into the curriculum not only enhances technical competence but also promotes a reflective and ethical perspective on healthcare practice. This study reinforces this assertion, demonstrating that students with a greater orientation toward USR also show a greater commitment to health equity. However, it has been noted that nursing ethics education remains anchored in traditional theoretical models, which limits its applicability to clinical decision-making [15]. In this sense, although the present study finds a positive correlation between USR and the development of ethical-religious competencies, their integration into clinical practice may require more experiential pedagogical strategies.

Statistical analysis revealed that students with higher levels of USR also showed a greater disposition toward ethical reflection and a proactive attitude toward sustainability challenges in health care. Correlation analysis indicated that for every standard deviation increase in the USR score, the development of ethical-religious competencies increased by 0.33 units (*p* < 0.001), while social sustainability competencies increased by 0.67 units (*p* < 0.001). Qualitative studies indicate that nursing programs that actively incorporate USR produce graduates with a greater commitment to community engagement and improved skills in addressing ethical dilemmas in clinical practice. While the need for community-based learning strategies to strengthen USR is often emphasized, the findings of this study suggest that, in the context of private universities in Santiago, these values can be effectively developed within academic training without necessarily relying on external experiences [54].

In addition, the results indicated that there are no significant differences in the levels of USR according to variables such as gender, age, or socioeconomic level (*p* > 0.05). This suggests that the training in USR in the private universities analyzed has been equitable, allowing students, regardless of their demographic characteristics, to internalize these values in an equivalent manner. However, a moderate correlation was found between participation in volunteer activities (F1) and higher levels of USR (r = 0.67, *p* < 0.001), indicating that active involvement in the community may further enhance the development of these values. A similar finding has been documented, showing that cultural competence is crucial in nursing education and plays a significant role in promoting health equity. However, there is no direct evidence that participation in volunteer activities and community projects leads to increased awareness of health equity and social justice [8]. The results of this study complement this perspective, indicating that while academic training in USR is effective, combining it with practical volunteer experiences can have an even greater impact on the consolidation of these values.

Despite these positive findings, this study also highlights the need to strengthen the practical application of USR in nursing programs. Although the results indicate a significant correlation between USR and the development of ethical and sustainability competencies, the long-term impact of these values on professional performance remains an area that requires further exploration. Previous studies have indicated that integrating strategies such as community-based learning and ethical dilemma simulations can enhance the practical application of these values in clinical practice [14,22]. The development of ethical competencies is a crucial component of nursing education, with legal regulations and institutional frameworks playing a key role in shaping ethics education. Integrating ethical training into undergraduate nursing programs is essential to prepare students for the moral complexities of clinical practice, particularly in managing moral suffering and ethical dilemmas in patient care. Ensuring that future nurses are equipped with strong ethical foundations enables them to navigate challenging situations with professionalism and integrity [14]. The findings of our study reinforce this idea, suggesting that, although training in USR contributes to the development of ethical values, their consolidation in professional practice could benefit from active methodologies that allow the direct application of these principles in real health care contexts.

### 4.1. Study Limitations

A key limitation of this study is its exclusive focus on private universities in Santiago, Chile, which limits the generalizability of the findings to other regions or public institutions. Although the sample of 400 students was sufficient for statistical analyses, future research could expand this study to a national level and consider including graduates to assess the long-term impact of USR training on professional performance. In addition, this study focused mainly on students’ perceptions and attitudes; therefore, longitudinal studies analyzing the evolution of ethical and sustainability competencies in nursing professionals throughout their careers are recommended.

### 4.2. Practical Recommendations

In terms of practical recommendations, the results suggest that the integration of USR in university education should continue to be strengthened. It is recommended that universities incorporate active methodologies, such as ethical dilemma simulations and community-based learning, to enable students to apply these values in real healthcare settings. Also, nursing programs should promote more opportunities for volunteering and participation in community projects, as this could enhance the development of ethical and sustainability competencies. Collaboration between universities and communities, as well as the creation of partnerships with non-profit organizations, should be encouraged.

## 5. Conclusions

This study addresses the factors that define the socially responsible behaviors of nursing students in private universities in Santiago (Chile), as well as the impact of sociodemographic variables on these behaviors and how these in turn influence their professional preparation.

First, the factors that define socially responsible behaviors of nursing students are mainly related to their orientation towards University Social Responsibility (USR). Results show that students with a greater awareness of USR tend to develop stronger ethical competencies, such as in-depth ethical analysis and a greater commitment to health equity and sustainability. These students not only have a greater ability to identify and address social challenges within the health field but also take an active stance towards promoting social justice in their professional practices.

Regarding the influence of sociodemographic variables, the study reveals that, despite diversity in factors such as age, gender, number of children, educational gratuity, nationality and ethnicity, there are no statistically significant differences in students’ socially responsible behaviors according to these variables. This suggests that, regardless of these personal characteristics, students in general show similar socially responsible behavior when they have a strong orientation towards USR. However, this also highlights the importance of intrinsic factors, such as personal values and academic training received, over sociodemographic variables in promoting ethical and responsible behaviors.

In terms of how these socially responsible behaviors influence their professional preparation, the results indicate that students who adopt USR principles in their training perform better in practical health-related situations. These behaviors enable them to be more effective in working with vulnerable populations, making decisions based on equity and social justice, and addressing sustainability issues in health care. Training in USR not only enhances technical competence, but also strengthens leadership and interdisciplinary collaboration skills, which are essential in the context of today’s health systems.

The development of socially responsible behaviors also has a direct impact on the employability of graduates, as these qualities are highly valued by employers in the health sector, who are looking for professionals who can integrate ethics, social justice, and sustainability into their daily practice. This approach not only improves the quality of care provided but also contributes to the creation of more humanized and sustainable models of care.

Consequently, universities can adopt a comprehensive approach to strengthen social responsibility in nursing students by combining practical experiences, innovative pedagogical approaches, and the incorporation of ethical values in all areas of their training. To effectively integrate social responsibility into education, educators must go beyond the theoretical teaching of ethical and social values by providing students with opportunities to apply them in real situations. These strategies will not only enhance the technical preparation of future professionals but also make them agents of change committed to equity and sustainability in health care. By integrating USR in a practical and continuous manner, future nurses will be better prepared to meet the ethical, social, and sustainability challenges that characterize today’s healthcare landscape.

In summary, the socially responsible behaviors of nursing students are defined by their orientation towards USR, rather than by sociodemographic variables. These practices, in turn, positively influence their professional preparation, improving their ability to address ethical and social challenges in health care. Integrating these principles into university education contributes to training not only technically competent nurses, but also professionals committed to the transformation of health systems towards more equitable and sustainable models.

## Figures and Tables

**Table 2:** Participant sample characterization.

Variable	Symbol	Category/Level	Frequency	Percentage
Age	(AGE)	Between 18 and 22 years old	268	79.5%
		Between 23 and 27 years old	58	17.2%
		28 or more years old	11	3.3%
Gender	(GEN)	Female	272	80.7%
		Male	63	18.7%
		Other	2	0.6%
Children	(CHI)	Yes	16	4.7%
		No	321	95.3%
Number of children	(NCH)	None	321	95.3%
		1 child	15	4.5%
		2 children	1	0.3%
Educational gratuity	(GRAT)	Yes	160	47.5%
		No	177	52.5%
Nationality	(NAT)	Chilean	330	97.9%
		Ecuadorian	1	0.3%
		Haitian	1	0.3%
		Peruvian	2	0.6%
		Other	3	0.9%
Native people	(NPE)	None	307	91.1%
		Mapuche	27	8.0%
		Aimara	3	0.9%

**Table 3:** Univariate descriptive statistical analysis.

Variables	N	Standard Deviation		Skewness		Kurtosis	
	Valid Number	Reported	Boero et al., 2020 [34]	Reported	Boero et al., 2020 [34]	Reported	Boero et al., 2020 [34]
SA1 *	336	1.372 *	1.169 *	−0.055 *	0.923 *	−1.254 *	−0.079 *
SA2 *	335	1.499 *	0.947 *	0.546 *	1.794	−1.203 *	2.623
SA3 *	332	1.494 *	1.055 *	0.011 *	1.540	−1.466 *	1.598 *
SA4 *	333	1.444 *	0.932 *	0.544 *	2.043	−1.137 *	3.572
SA5 *	335	1.553 *	1.206 *	−0.404 *	0.907 *	−1.392 *	−0.222 *
CR1	336	0.845 *	0.774 *	−3.478	−3.048	11.800	9.780
CR2	336	0.799 *	1.213 *	−3.118	−1.396	10.219	0.840 *
CR3	337	0.900 *	0.958 *	−1.770	−0.746 *	3.566	0.185 *
CR4	337	0.832 *	0.777 *	−2.585	−2.580	6.963	7.098
CR5	337	0.737 *	0.692 *	−3.422	−1.968	13.053	4.420
CR6	336	0.756 *	0.784 *	−3.544	−2.380	13.378	6.227
CR7 *	336	1.142 *	1.269 *	−0.549 *	0.128 *	−0.455 *	−0.989 *
CC1 *	335	1.078 *	1.079 *	−0.814 *	−0.714 *	0.101 *	−0.203 *
CC2 *	336	1.411 *	1.150 *	0.410 *	1.601	−1.162 *	1.508 *
CC3 *	336	1.315 *	1.497 *	−1.127 *	0.499 *	0.045 *	−1.201 *
CC4 *	335	1.163 *	1.084 *	−1.012 *	−0.692 *	0.320 *	−0.166 *
CC5 *	336	1.416 *	1.317 *	−0.521 *	0.243 *	−1.062 *	−1.061 *
RE1 *	253	1.553 *	1.228 *	0.104 *	1.923	−1.549 *	2.279
RE2 *	239	1.477 *	1.186 *	0.270 *	1.970	−1.34 8*	2.559
RE3 *	225	1.352 *	0.829 *	0.880 *	3.141	−0.400 *	9.357
RE4 *	239	1.360 *	1.074 *	0.404 *	1.884	−0.981 *	2.446
SW1	335	0.770 *	0.491 *	−3.274	−2.937	11.676	12.022
SW2	335	0.840 *	0.673 *	−2.430	−1.095	6.784	1.269 *
SW3	337	0.870 *	0.853 *	−1.849	−1.006	4.263	0.799 *
SW4	337	0.903 *	0.816 *	−1.829	−1.034	3.613	1.140 *
N valid (per list)	207						

* Variables that satisfy the pre-established parameters of standard deviation, skewness, and kurtosis.

**Table 4:** Anti-image correlation.

	SA1	SA2	SA3	SA4	SA5	CR7	CC1	CC2	CC3	CC4	CC5	RE1	RE2	RE3	RE4
SA1	0.852 ^a^	−0.138	−0.145	−0.039	−0.085	−0.137	0.053	−0.090	0.024	−0.027	−0.134	−0.019	0.167	0.009	−0.172
SA2	−0.138	0.880 ^a^	−0.260	−0.230	−0.059	−0.117	−0.024	−0.092	0.010	−0.031	0.051	−0.111	0.063	0.055	−0.063
SA3	−0.145	−0.260	0.850 ^a^	−0.210	−0.337	.087	−0.025	−0.183	0.089	−0.047	−0.046	−0.015	−0.059	0.001	0.078
SA4	−0.039	−0.230	−0.210	0.881 ^a^	−0.179	−0.077	0.052	−0.138	0.034	0.041	0.019	0.060	−0.102	−0.111	0.073
SA5	−0.085	−0.059	−0.337	−0.179	0.869 ^a^	.067	0.061	0.097	−0.118	−0.067	−0.145	−0.022	0.009	−0.002	−0.013
**CR7**	−0.137	−0.117	0.087	−0.077	0.067	0.669 ^a^*	−0.111	0.050	0.048	−0.156	−0.084	−0.005	0.061	−0.140	0.093
CC1	0.053	−0.024	−0.025	0.052	0.061	−0.111	0.830 ^a^	−0.051	−0.164	−0.204	−0.103	0.017	0.003	−0.070	−0.002
CC2	−0.090	−0.092	−0.183	−0.138	0.097	0.050	−0.051	0.861 ^a^	−0.117	−0.069	−0.207	0.123	−0.008	−0.062	−0.017
CC3	0.024	0.010	0.089	0.034	−0.118	0.048	−0.164	−0.117	0.756 ^a^	−0.375	−0.121	0.037	−0.012	0.000	0.000
CC4	−0.027	−0.031	−0.047	0.041	−0.067	−0.156	−0.204	−0.069	−0.375	0.804 ^a^	−0.105	−0.016	−0.019	0.096	−0.053
CC5	−0.134	0.051	−0.046	0.019	−0.145	−0.084	−0.103	−0.207	−0.121	−0.105	0.907 ^a^	0.004	−0.112	−0.040	0.019
RE1	−0.019	−0.111	−0.015	0.060	−0.022	−0.005	0.017	0.123	0.037	−0.016	0.004	0.801 ^a^	−0.705	−0.068	−0.127
RE2	0.167	0.063	−0.059	−0.102	0.009	0.061	0.003	−0.008	−0.012	−0.019	−0.112	−0.705	0.785 ^a^	−0.192	−0.240
RE3	0.009	0.055	0.001	−0.111	−0.002	−0.140	−0.070	−0.062	0.000	0.096	−0.040	−0.068	−0.192	0.876 ^a^	−0.454
RE4	−0.172	−0.063	0.078	0.073	−0.013	0.093	−0.002	−0.017	0.000	−0.053	0.019	−0.127	−0.240	−0.454	0.866 ^a^

^a^ Measure of Sampling Adequacy (MSA); *: minimum value on the main diagonal of the matirx.

**Table 5:** Communalities.

Variable	SA1	SA2	SA3	SA4	SA5	CC1	CC2	CC3	CC4	RE1	RE2	RE3	RE4
Initial	0.277	0.400	0.539	0.424	0.413	0.200	0.299	0.320	0.364	0.798	0.838	0.675	0.712
Extraction	0.282	0.470	0.677	0.500	0.429	0.267	0.322	0.490	0.556	0.794	0.889	0.688	0.734

**Table 6:** Exploratory factor analysis for three factors.

KMO and Bartlett’s Test			
Kaiser–Meyer–Olkin Measure of Sampling Adequacy.		0.831	
Bartlett’s Test of Sphericity	Approx. Chi-Square	1318.641	
	Degree of freedom	78	
	Significance	0.000	
**Pattern Matrix ^a^**			
**ID**	**Factor 1**	**Factor 2**	**Factor 3**
SA3	0.837		
SA4	0.705		
SA2	0.680		
SA5	0.612		
SA1	0.535		
CC2	0.458		
RE2		−0.946	
RE1		−0.893	
RE4		−0.847	
RE3		−0.809	
CC3			0.708
CC4			0.707
CC1			0.517
Eigenvalue	4.067	1.978	1.051
% of Variance	31.286	15.219	8.087
Cumulative %	31.286	46.504	54.591
**Factor Correlation Matrix ^b^**			
Factor	1	2	3
1	1.000	−0.309	.342
2	−0.309	1.000	−0.132
3	0.342	−0.132	1.000

^a^ Extraction Method: Unweighted Least Squares. Rotation Method: Oblimin with Kaiser Normalization. Rotation converged in six iterations. ^b^ Extraction Method: Unweighted Least Squares. Rotation Method: Oblimin with Kaiser Normalization.

**Table 7:** Confirmatory factor analysis for two factors.

KMO and Bartlett’s Test		
Kaiser–Meyer–Olkin Measure of Sampling Adequacy.		0.849
Bartlett’s Test of Sphericity	Approx. Chi-Square	1670.200
	Degree of freedom	45
	Significance	0.000010
**Rotated Loading Matrix**		
**Variable (Item)**	**Factor 1 (F1)**	**Factor 2 (F2)**
Factor Name	Social Sustainability	Ethical-Religious
SA1	0.585	
SA2	0.741	
SA3	0.874	
SA4	0.763	
SA5	0.679	
CC2	0.559	
RE1		0.945
RE2		0.973
RE3		0.888
RE4		0.899
% of Variance	47.288	23.679
Cumulative %	47.288	70.967
Eigenvalue	4.72883	2.36791
% Eigenvalue	67%	33%
Factor Correlation Matrix		
Factor	F1	F2
F1	1.000	
F2	0.346	1.000

**Table 8:** Validation and reliability reported in previous articles and parameters.

Article	Country	Sample	Method	Factors	MIF	χ^2^/df	RMSEA	AGFI	GFI	CFI	NNFI	RMSR
Boero et al. [34]	Chile	1935	EFA/CFA	5	4	4.11	0.057 *	NR	NR	0.960 *	0.991 **	NR
Proposed Model	Chile	337	EFA/CFA	2	4	1.14 **^,+^	0.026 **	0.995 **	0.997 **	0.998 **	0.997 **	0.030 **
Schermelleh-Engel et al. [44]	Parameters	≥200	Good fit	-	NR	≥0 ≤2	≤0.05	≥0.90 ≤1.00	≥0.95 ≤1.00	≥0.97 ≤1.00	≥0.97 ≤1.00	<0.05 ^++^
			Acceptable fit	-	≥3	>2 3	>0.05 ≤0.08	≥0.85 <0.90	≥0.90 <0.95	≥0.95 <0.97	≥0.95 <0.97	≥0.05 ≤0.08 ^++^

NR: not reported. ** Good fit; * acceptable fit. ^+^ Robust Mean and Variance-Adjusted Chi Square. ^++^ indicated in Kalkan et al. [48].

**Table 9:** Reliability statistics.

Scale	Valid cases	Number of Items	Cronbach’s Alpha
Factor 1	330	6	0.800
Factor 2	221	4	0.933
ICOSORE-U-10	216	10	0.841

**Table 10:** Cross Tables characterization variables and ICOSORE-U-10 (symmetric measures).

Cross-Table	Test		Value	Asymptotic Standardized Error ^a^	Approximate T ^b^	Approximate Significance
Age * ICOSORE-U-10	Ordinal per ordinal	Kendall’s Tau-C	−0.026	0.047	−0.557	0.578
		Gamma	−0.075	0.135	−0.557	0.578
	N of valid cases		216			
GEN * ICOSORE-U-10	Ordinal per ordinal	Kendall’s Tau-C	0.029	0.045	0.653	0.514
		Gamma	0.085	0.130	0.653	0.514
	N of valid cases		216			
CHI * ICOSORE-U-10	Ordinal per ordinal	Kendall’s Tau-C	0.036	0.030	1.181	0.238
		Gamma	0.334	0.258	1.181	0.238
	N of valid cases		216			
NCH * ICOSORE-U-10	Ordinal per ordinal	Kendall’s Tau-C	−0.036	0.030	−1.181	0.238
		Gamma	−0.334	0.258	−1.181	0.238
	N of valid cases		216			
GRA * ICOSORE-U-10	Ordinal per ordinal	Kendall’s Tau-C	−0.093	0.073	−1.280	0.201
		Gamma	−0.132	0.102	−1.280	0.201
	N of valid cases		216			
NAT * ICOSORE-U-10	Ordinal per ordinal	Kendall’s Tau-C	0.014	0.014	0.987	0.323
		Gamma	0.319	0.285	0.987	0.323
	N of valid cases		216			
NPE * ICOSORE-U-10	Ordinal per ordinal	Kendall’s Tau-C	−0.012	0.039	−0.300	0.764
		Gamma	−0.052	0.173	−0.300	0.764
	N of valid cases		216			

^a^ The null hypothesis is not assumed. ^b^ Use of the asymptotic standard error that presupposes the null hypothesis.

## Data Availability

Data are available in Supplementary Materials.

## Supplementary Materials

The following supporting information can be downloaded at: https://www.mdpi.com/article/10.3390/nursrep15030093/s1, ICOSORE-U_1-337_v2.xlsx (data), ICOSORE_U_337_13.dat.txt (data, 13 items), ICOSORE_U_337_10.dat.txt (data, 10 items), ICOSORE_U_LAB.txt (Label, 13 items), ICOSORE_U_LAB_10.txt (Label, 10 items), output_FACTOR13.txt (Output, 13 items), output2F10I.txt (Output, 10 items).

## Public Involvement Statement

There was no public involvement in any aspect of this research.

## Guidelines and Standards Statement

This manuscript was drafted against the Recommendations for reporting the results of studies of instrument and scale development and testing [55] for psychometric testing research.

## Abbreviations

The following abbreviations are used in this manuscript:

USR	University Social Responsibility
SDG	Sustainable Development Goal
4Ps	Pathophysiology, Pharmacology, Physical/Health Assessment, and Health Promotion
ICOSORE-U	Inventory of Socially Responsible Behaviors in University Students (in Spanish).
EFA	Exploratory Factor Analysis
CFA	Confirmatory Factor Analysis
ULS	Unweighted Least Squares
RULS	Robust Unweighted Least Squares
MSA	Measure of Sampling Adequacy
MIF	Minimal Number of Items Per Factor
RMSEA	Root Mean Square Error of Approximation
AGFI	Adjusted Goodness-of-Fit Index
GFI	Goodness-of-Fit Index
CFI	Comparative Fit Index
NNFI	Non-Normed Fit Index
RMSR	Root Mean Square Root of Residuals
GEN	Gender
CHI	Children
NCH	Number of children
GRAT	Educational gratuity
NAT	Nationality
NPE	Native people
SA	Social Assistance
CR	Care Respect
CC	Cultural Citizenship
RE	Religion
SW	Study Work
KMO	Kaiser–Meyer–Olkin
